# Severe secondary hyperparathyroidism: an increasing problem in CKD but the best management option is still unknown

**DOI:** 10.1590/2175-8239-JBN-2024-E004en

**Published:** 2024-03-15

**Authors:** Mark Kung Dah Tiong, Nigel David Toussaint

**Affiliations:** 1 The Royal Melbourne Hospital, Department of Nephrology, Parkville, Australia.; 2 University of Melbourne, Department of Medicine, Parkville, Australia.

Hyperparathyroidism is a near ubiquitous feature of advanced stages of chronic kidney disease (CKD), developing as a secondary response to phosphate retention and abnormalities in calcium and vitamin D metabolism^1,2^. The most explicit clinical manifestation is bone disease, largely driven by chronic changes in bone remodeling, and resulting in increased risk of fracture and associated morbidity. However, secondary hyperparathyroidism (SHPT) is also a key component in the pathogenesis of chronic kidney disease-mineral and bone disorder (CKD-MBD), and is linked to the development of accelerated cardiovascular calcification and cardiovascular mortality^1^.

Severity of SHPT generally parallels the progression of CKD and is worst in those who reach kidney failure being treated with dialysis^2^. However, while kidney transplantation ostensibly restores many of the underlying drivers of SHPT, many kidney transplant recipients also remain at high risk of bone abnormalities^3^. This is largely attributable to the residual effects of years of CKD-MBD-related bone disease before transplantation as well as the superimposed effects of immunosuppression, largely related to corticosteroid use (albeit this has been partially abrogated by modern steroid-minimizing protocols)^3^. In addition to these factors, residual hyperparathyroidism post-transplantation may also compound fracture risk, and persistent hyperparathyroidism is linked to increased risk of transplant allograft dysfunction^4^. These factors have fueled the development of therapeutic interventions that directly target SHPT in dialysis and transplant patients.

In addition to dialysis and transplantation itself, interventions to manage SHPT have included intestinal phosphate binders, vitamin D and its analogs, surgical parathyroidectomy, and more recently, calcimimetics such as cinacalcet. While each of these interventions have been shown to lower serum parathyroid hormone (PTH), the optimum order and timing of their use remains unclear^1^. As a result, selection of these therapies is largely driven by local availability, clinician preference, and patient choice.

In this journal, Ramos et al.^5^ present a single-center, retrospective analysis of patients with SHPT who have kidney failure, either being treated with maintenance hemodialysis or were recipients of a kidney transplant (from a tertiary referral center with a dedicated CKD-MBD clinic). The authors compared three main treatment strategies: supportive measures alone (adjustments to dialysis, use of phosphate binders, and vitamin D-based compounds), or supportive measures with the addition of either cinacalcet or parathyroidectomy. They observed that each of these three strategies was associated with significant reductions in serum PTH over a 12-month observation period. However, among dialysis patients who underwent a parathyroidectomy, a larger reduction in serum PTH was seen compared to those who did not, and this group was also more likely to achieve a nominal PTH target of ≤ 300 pg/mL at 12 months. Among transplant recipients, the difference between treatment approaches was less pronounced, with each treatment strategy producing significant, but overall similar reductions in serum PTH. A slightly larger proportion of transplant recipients who underwent a parathyroidectomy achieved a nominal target of PTH ≤ 100 pg/mL with normal ionized calcium (90% versus 80% and 76% in the post-transplant supportive therapies and cinacalcet groups respectively). Interestingly, the vast majority of patients excluded from the study (206 patients without SHPT out of 402 patients undergoing follow-up in the CKD-MBD clinic) had had a previous parathyroidectomy.

As Ramos et al.^5^ outline, with more liberal PTH targets according to current international clinical guidelines^1^, an increasing proportion of CKD patients may develop more severe SHPT refractory to medical therapy (including treatment with calcimimetics). Whether parathyroidectomy is the ‘best choice’ for the management of severe SHPT as described in their conclusion is yet to be determined; and as the authors themselves acknowledge, their study has inherent limitations due to its retrospective observational design. It is unclear how patients were assigned through clinical practice to each treatment group (clear bias by indication) and what (if any) interventions had been tried prior to the study period. As the authors discuss, in their setting and in Brazil in general, there is limited access to cinacalcet and parathyroidectomy, which would be expected to influence selection of individuals who receive these treatments. This could have resulted in imbalances between the analyzed groups, and likely accounts for differences in baseline demographics, including the parathyroidectomy group being younger and having higher baseline levels of PTH^5^. Arguably though, the parathyroidectomy group with evidence of more severe SHPT at baseline does not necessarily detract from their main contention of surgical intervention being associated with more significant reductions in serum PTH. In fact, as the authors suggest, this may actually enhance the argument. Further, their results are broadly consistent with previous reports^1^ and add real-world data from the relatively under-represented South American region.

What is harder to discern though is whether the more pronounced reductions in PTH with parathyroidectomy reported by Ramos et al.^5^ and other studies translates into superior patient-centered outcomes, especially with only one-year follow up reported. Given that SHPT seems to be directly correlated with patient risk of fracture and cardiovascular outcomes in CKD, use of serum PTH as a surrogate treatment target is attractive. Notably however, there is a paucity of evidence to confirm that therapeutic lowering of PTH in CKD results in demonstrable improvements in patient-level outcomes, despite convincing evidence that interventions such as vitamin D agents^6^ and calcimimetics^7^ lower serum PTH in CKD.

This is not to argue that efforts to manage SHPT are futile, but that clinicians should be cognizant of the limitations of our current data when discussing treatment options with their patients. For instance, whether pursuing further reductions in PTH from parathyroidectomy compared to non-surgical options will yield additional benefits that outweigh potential harms of surgery remains unclear. Further, introduction of the next generation of calcimimetics^8,9,10^ that promise higher efficacy and better tolerability than cinacalcet may make this landscape more challenging for clinicians and patients to navigate. Ultimately, all of this highlights the need for well-designed randomized trials with follow up of long enough duration to properly evaluate potential benefits (and harms) of these treatment strategies.
